# Usability of Public Play Spaces for Children with Disabilities

**DOI:** 10.1155/2023/4306627

**Published:** 2023-12-29

**Authors:** Asma Nidaul Haq, Yuko Ito, Natsuka Suyama, Peter Bontje, Hana Hanifah, Kaoru Inoue

**Affiliations:** ^1^Department of Occupational Therapy, Graduate School of Human Health Sciences, Tokyo Metropolitan University, Tokyo, Japan; ^2^Department of Humanities, Illinois Institute of Technology, Chicago, IL, USA

## Abstract

**Purpose:**

To investigate the usability of public play spaces for children with disabilities by exploring their experiences in accessing and using these spaces and to further discuss recommendations for designing such spaces that are usable for these children.

**Methods:**

A hermeneutic phenomenological approach was employed to explore the experience of children with disabilities regarding the public play spaces. Seven children and their caregivers from two inclusive elementary schools in Central Java Province, Indonesia, participated in the study. Online semistructured interviews with the children were held using Zoom, followed by telephonic interviews with their parents. In addition, video recordings of the observation of the children's participation in the play spaces were gathered. The interview and video observation data were analyzed using van Manen's hermeneutic phenomenology thematic analysis method.

**Results:**

Five themes arose regarding the experiences of children with disabilities of accessing and using the public play spaces: (1) where time appeared to speed up, (2) “I like the tall one … I like extreme,” (3) fostering connectedness, (4) the need for a safe space, and (5) how a play space should be.

**Conclusion:**

The public play spaces have meaningful values for the children with disabilities and their family, as they offered the opportunities to play, explore, interact with friends and families, enjoy nature, interact with animals, and learn. However, it is essential to provide a safe space in which children are free from physical and emotional harm, so that they can fully participate with confidence and a sense of autonomy. Nevertheless, it was also found that children, regardless of their abilities, craved risky and challenging play opportunities. This study also highlights the necessity of awareness-raising intervention programs to foster the inclusion of children with disabilities in public play space settings.

## 1. Introduction

Play is a fundamental right of all children, regardless of their abilities [1]. Moreover, it is their most important occupation, vital for their development, health, and quality of life [2, 3]. Outdoor play has been shown to have beneficial effects on health and social, emotional, cognitive, and physical skill development, as well as academic capabilities [4]. Public play spaces, such as community parks and playgrounds, are common settings for children to engage in play [5]; they are greatly valued by children, including those with disabilities, and their families [6, 7]. These spaces provide them the opportunity to meet, play, and interact with each other [8, 9].

Despite the importance of play in children's lives, there has recently been an increased concern regarding children's limited access to outdoor play spaces that is caused by numerous potential factors, such as the unappealing and inaccessible outdoor play environments, more interest in screen-based activities, increased urbanization, and busy personal and professional lives [10, 11]. For children with disabilities, however, the issue is worsened because they tend to experience additional challenges including physical, social, and attitudinal barriers that result in limited accessibility and usability and reduced participation in the public play spaces [6, 7, 12].

Being unable to engage in play, which is a meaningful occupation for children, is a threat to their occupational right to develop “through participation in occupations for health and social inclusion” (p. 81) [13]. Therefore, it becomes an occupational injustice, with the possibility of leading to occupational deprivation, when children are declined the opportunity to play. Since occupational therapists are equipped with knowledge about human occupations, disabilities, and the environment, it is one of our duties to advocate for children's right to play and address issues of occupational injustice [14]. Likewise, occupational therapists' roles are expanding beyond a focus on curative and preventative treatment of individuals toward the right of everyone to engage in and enrich engagement and participation in occupations of interest [15].

Previous studies have relied predominantly on the accounts of adult caregivers as a proxy for exploring children's perspectives on play spaces [16]. Nevertheless, it is essential to include children as informants when designing for play [17], as there may be discrepancies in how they experience play activities and how these experiences are viewed by adults [18]. In addition, previous studies hitherto conducted are limited to high-income, Western countries. This study was conducted in Indonesia, which is classified as upper-middle-income country in 2022. Hence, the aim of the present study is twofold. Firstly, it attempted to investigate the usability of the community parks and playgrounds for children with disabilities by exploring their experiences regarding their accessibility and usability from the perspective of children and their primary caregivers. The term *usability* itself refers to the ability to access and utilize the environment equally; it transcends accessibility as it embraces the individuals' subjective evaluations of performing an activity within an environment, instead of merely focusing on official standards and guidelines [19]. Secondly, it sought to discuss future recommendations regarding a playground design that is usable for children with disabilities.

## 2. Methods

### 2.1. Design

To gain an in-depth understanding of the experience of children with disabilities regarding public play spaces, this study employed a hermeneutic phenomenological approach. Phenomenology, in essence, is the study of lived experiences through experiential accounts [20]. Phenomenological inquiry asks the question of “What is this experience like?” as it seeks to unravel the meanings as they are perceived in everyday life [21]. Phenomenology focuses on elucidating details and seemingly frivolous aspects of the experience that may have been taken for granted [22]. It is essential to investigate the experience as it is lived, rather than as conceptualized by the researchers [20]. Whereas empirical or psychological phenomenology focuses on participants' description of the experiences [23] to obtain “a near real picture” [24], hermeneutic phenomenological research is interpretive in its methods and focus, in which the researcher interprets the meaning of the lived experiences to gain a deeper understanding [20, 25]. Hermeneutic phenomenology emphasizes the “meaning of the meaning of the text,” namely, the psychological implications of the “speech,” “language,” or “set of words” in a specific context [20, 25].

In qualitative studies, the researcher's perspective is fundamentally interwoven with the research processes, and it is important to clarify how it shapes the inquiry [26]. At the time of the study, the main author was a master's degree student at a University Department of Occupational Therapy. She is of Indonesian origin and used to work with elementary school students with developmental disabilities in Indonesia. As an occupational therapist who works with children, she highly regards the importance of play in children's lives, which is believed to be both a means and an end. Exclusion, marginalization, and strong prejudices experienced by children with disabilities have been a significant concern [27]. In Indonesia, despite the existence of laws and policies concerning the promotion of the rights of people with disabilities, ensuring their enforcement has been inadequate [27]. The law itself perpetuates stigma as individuals with disabilities are defined as members of society who have problems and social dysfunction [28]. The main investigator of this study holds the belief that occupational therapists, among others, are responsible in promoting inclusion and participation of individuals with disabilities.

### 2.2. Participants and Recruitment

Using a purposive sampling method, seven children with disabilities enrolled in two inclusive elementary schools in Central Java Province, Indonesia, including their parents/primary caregivers participated in this study. Central Java Province was the main author's birthplace, where she had easier access to and was most familiar with the population. The inclusion criteria were children with disabilities aged 7-12 years who have cognitive and communication abilities to understand and answer simple questions (as judged by qualified school professionals). Maximum variation sampling was employed to capture diverse perspectives, which is ideal in qualitative research [29]. In phenomenological (empirical) and hermeneutic phenomenological studies, the purpose of participant diversity is to increase the prospect of rich and unique stories of the experience, to attain a deeper understanding about the phenomenon [30, 31].

The recruitment was conducted with the help of the headmasters of the institutions. Following a discussion with the headmasters regarding suitable participants, the prospective participants were referred by the headmasters and subsequently contacted by the main author. All referred participants agreed to participate.

### 2.3. Ethical Considerations

This research was carried out with the approval of the research ethics committee of the graduate school to which the author belongs. The headmasters of the institutions agreed to collaborate before contacting the participants. The participants were informed about the study's purpose and nature and the voluntary nature of participation and that they could refuse to participate or withdraw from the study at any time without any repercussions. The researcher reassured the participants about the confidentiality of their responses and the protection of their personal information. Finally, informed consent was obtained from the primary caregivers and the children. The audio and video recordings were carried out with the permission of the participants.

### 2.4. Data Collection

Online semistructured interviews with the children were held through Zoom and were video recorded. Multiple strategies were utilized to reinforce the children's active engagement in the interview process, such as using photographs as cues/prompts to stimulate a conversation about their experiences and help them recall events [32] and drawing to facilitate the children in expressing their views and feelings [33]. Moreover, drawing is regarded as a fun and calming activity and was also utilized to foster rapport building [29]. During the interviews, some of the children were instructed to draw their experiences and explain them afterwards. Additionally, semistructured interviews were employed, as opposed to an especially open, unstructured interview, because some of the children required some careful prompting, to ensure that the breadth of the topics was covered [34]. To establish a good rapport, multiple interviews were held that began with asking the children general questions, including about their daily routine and favorite play activities, that they might find easier to answer [29]. Such questions could function as icebreakers and allow the researcher to learn and attune to the communication style of the child [35]. The children were asked open-ended questions (e.g., tell me what it is like when you play on the parks/playgrounds); through subsequent questions, the researcher attempted to explore their feelings and contexts (Appendix A). Furthermore, the interviews lasted for 15-30 minutes. The short-duration interviews were preferred considering children's shorter attention span. Additionally, during the interviews, a teacher aide to whom each child was closest and most familiar was present to provide support and comfort. The assistant teachers were instructed not to give answers on behalf of the children, nor to influence or steer the children's answers.

Prior to the semistructured interviews with children, the main author collaborated with participating institutions to conduct nonparticipant observations of the children's engagement in the public play spaces that they commonly visited. Each child was followed and recorded by an appointed teacher who maintained a reasonable distance that allowed for a clear view of the child's activities and an audible voice without disrupting the child's movements. To facilitate note recording, an observation guide was developed (Appendix B). In addition to the observations and the interviews with the children, telephonic unstructured interviews with primary caregivers were carried out, except for one caregiver who was not available for interview due to illness and scheduling conflicts during the data collection period. All interviews were performed by the first author.

### 2.5. Data Analysis

The interview transcriptions and the descriptive notes from the observations were analyzed by the main investigator using van Manen's hermeneutic phenomenology thematic analysis method, which van Manen himself referred to as “hermeneutic phenomenological reflection” (p. 77) [20]. The primary notion behind it is to identify the essential meaning of a phenomenon. Being generally less structured compared to the other phenomenological analysis methods, three approaches could be considered to isolate thematic statements: (1) *the holistic or sententious approach*, in which the researcher looked at the text as a whole and wrote a “sententious phrase” that might capture the underlying meaning of the text; (2) *the selective or highlighting approach*, where the researcher read the text several times and highlighted seemingly significant statements and phrases that might be revealing about the experience; and (3) *the detailed or line-by-line approach*, where the researcher reviewed every sentence or sentence cluster and asked “What does this sentence or sentence cluster reveal about the phenomenon or experience being described?” (pp. 92-93) [20]. Subsequently, the researcher gathered all the sentences or sentence clusters, followed by the thematic statement that represented their meanings in a Microsoft Word document. Consequently, the commonalities or possible commonalities (recurring themes) were identified and given appropriate phrases that represented the meaning of the themes [20]. The thematic statements were then organized and categorized as potential main themes. The data analysis stage was an iterative process that involved constant reflection and repeated reexamination and recategorization. Through graduate school seminars, peer debriefing sessions were held to discuss and review the development and categorization of the themes that resulted in the identification of the main themes. Among the seminar members was the study's third coauthor, a university professor proficient in qualitative data analysis and study who had published a number of qualitative research articles.

### 2.6. Rigor/Trustworthiness

To enhance the rigor and trustworthiness of this study, several strategies were employed. The triangulation of multiple data sources was performed to corroborate emerging findings [36]. To enable the transferability of the findings, rich and comprehensive descriptions about the participants and the themes were written [37]. Additionally, throughout the course of the research, peer debriefings were conducted regularly, involving graduate students and professors, among whom were experienced qualitative researchers. Finally, the main researcher maintained a reflective log to be aware of her experiences, thoughts, and assumptions and to avoid imposing her own perceptions in the research.

## 3. Results

Overall, seven children with disabilities and six caregivers participated in the interview.  Table 1 presents the demographic characteristics of the participants. A pseudonym was given to each child to protect the privacy of their identity. The data analysis uncovered five main themes: (1) where time appeared to speed up, (2) “I like the tall one … I like extreme,” (3) fostering connectedness, (4) the need for a safe space, and (5) how a play space should be.

### 3.1. Theme 1: Where Time Appeared to Speed Up

Public play spaces served multiple different meanings and purposes for each child. Nonetheless, fun was what the children were searching for. The children used the opportunity of being in these spaces to try different play equipment, play games with their friends, walk around observing nature and enjoying the fresh air, pick fruits from the trees, interact with animals, and so on. For instance, Ahmad described during an interview:

*In Kereta Park, I waved at the train driver, played on the swing, saw monkey, and bought a lot of street food. There was a fishpond, I watched the passing trains, watched windmill under the water, there were also miniature ships. While waiting for the train to pass sometimes I bought street food. If the train is approaching, I must quickly climb onto the spot to wave at the train driver.*

whereas Zaki mentioned:

*I prefer walking around while breathing fresh air. Because it's healthy for the body, and I could see the scenery, there's fish, there's Korean food.*

Correspondingly, the children looked forward to having their parents take them to parks or playgrounds, and they would spend hours engaging, only to feel that they had not played enough and that time was slipping away. When having fun, time seems to fly by, as insinuated in the statements made by Aldi and Vian's mother:

*[Q: whom do you usually play with?] Well, many friends. The sky suddenly got dark without me realizing.* (Aldi)

*Vian likes to go to the park with his mother and younger sister. ... He enjoyed playing so much that he didn't realize the passing time and still felt not enough.* (Vian's mother)

It is surmised that being in such a wide-open space might have rendered the children feeling more sense of independence and freedom than they would have otherwise felt in different settings. These environmental characteristics of the play spaces acted as affordances that invited children, as naturally curious creatures, to use their senses to explore and observe the things they encountered. In their exploration, they simultaneously engaged in creative pursuits where they ceaselessly attempted to discover fun things inherent to the surroundings, which was demonstrated, for instance, in Fafa's observational video:

*Fafa mischievously stomped hard and repeatedly on the metal bridge, resulting in an ear-piercing loud banging noise, the two other children immediately imitated Fafa's action. Having crossed the bridge, the trio giggled in satisfaction.*

**Figure 1:**
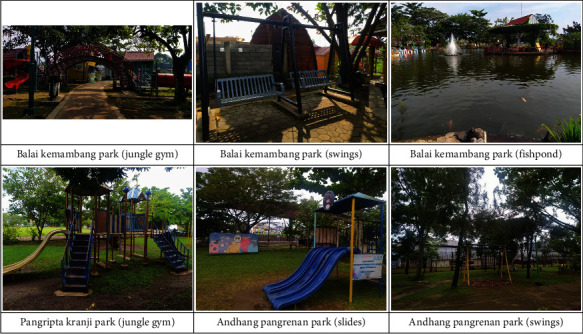
Public play spaces commonly visited by the participants.

During an interview, Zaki—a boy with autism spectrum disorder—when instructed to draw something related to his park experience, drew a fence, a slide, a trampoline, and a swing. Likewise, in the public play spaces visited by the participants in this study, the commonly provided play equipment were swings, slides, and jungle gym. Some of the bigger play spaces also offered rented toys/games, such as scooters and water bikes. Each child had their own favored play activities. Photos of the public play spaces where the observations took place are shown in Figure 1.

In addition, drawing from the children's interviews, the topic of animal encounters was brought up with enthusiasm by nearly all the children in this study when they were speaking about their park experience. Their interaction with animals (fish, wild birds, bees, monkeys, etc.) seemed to leave a lasting impression on most children. As exemplified by the observational video, Abdul's excitement about seeing fish was evident:

*Hehee what is there…? [walking with a wide stride, a puffed-out chest, and arms swinging back and forth]. [Pointing at the pond, he shouted], “Fish!” [as he jumped in excitement, spreading both arms high to the sides].*

### 3.2. Theme 2: “I Like the Tall One … I Like Extreme”

In their pursuits of fun, the children commonly sought activities that contained elements of riskiness or challenge. They were exhilarated by greater height, speed, complexity, or a trace of danger. Conversely, activities that were lacking in challenge were deemed uninteresting. Ergo, it was demonstrable that risk and challenge were important prerequisites of fun. Some of the children required words of encouragement or physical assistance when faced with challenges, but once overcome, they were observed beaming from success—feeling capable and confident.

For instance, Fafa's video showed his initial apprehension with the jungle gym where he climbed up a large steep bridge. However, as the other children could smoothly go through the obstacles, his teacher aide reassured him and promised to assist. Taking each step cautiously, he managed to go past the most challenging segment of the jungle gym and finally slid down the spiral slide, the last obstacle. During the subsequent interviews, Fafa indicated that he enjoyed this play equipment; thus, the jungle gym shifted from being something that initially inspired fear to becoming one of his favorite pieces of play equipment.

*[Q: Do you like this one (a picture of car rental arena shown on the screen)?] No, I like the previous one, the tall one (the jungle gym). … I was climbing that one (the jungle gym). I was not scared anymore, now I can climb that (grinning). But at that time, in the beginning, I was scared that I'd fall.* (Fafa, child interview)

The following remarks stated by Vian's mother and Ahmad further exemplified the children's keenness for risk and challenge:

*… Vian prefers games that are challenging and complex. He enjoyed playing so much that he didn't realize the passing time and still felt not enough.* (Vian's mother)

*[Q: what do you think about this slide?] Not long enough, I like it more extreme.* (Ahmad)

### 3.3. Theme 3: Fostering Connectedness

The public play spaces also functioned as places where children could interact. For some of the children, the prospect of being with peers was motivating. They often associated their park/playground experience with play partners. The observation videos revealed how the children were teasing each other, creating playful competitions or races, imitating and learning from one another, relaxing together, and so forth. The following are excerpts of the observational video description:

*Aldi and Ahmad could be seen among the six children crowding around on the semicircular bridge of the jungle gym. As they raced toward the spiral slide, they shrieked and laughed, creating their own little commotion. One followed by another arrived at the spiral slide, four children in a row sat on the slide, with one of them yelling a “ONE… TWO… THREE!”, they slid down together.*

*Alia, Abdul and Fafa sat leisurely on the merry-go-round, no one was holding the steering wheel. Alia rested her back on the backrest as she gulped down a bottle of tea beverage. Next to her, Abdul was also leaning his back against the backrest, sipping his bottle of tea occasionally. Fafa commented on how Alia almost finished her beverage, said she won against Abdul who still had more than half of his. Fafa took out his bottle from his backpack and started to drink too. The three looked comfortable and relaxed.*

Furthermore, as suggested by the respondents, public play space visits also served as a family recreational activity. The parents often took their children to parks on the weekends, holidays, or other special occasions, where they spent quality time together as a family. For example, Zaki's mother and Aldi's father recounted:

*Zaki treated the whole family to riding the pedal train and he became the driver. After that he also played with the shining balloons with his older and younger siblings. Then he bought street food using his own money. He's now able to keep his own money and wallet, so he doesn't have to ask for money from me.* (Zaki's mother)

*… if we visit Aldi's grandparents' house, I make time to visit Andhang Pangrenan (a public play space). … Aldi would ask me to allow him to ride a mini rickshaw around the park with his younger siblings. … When Aldi felt hungry, he would approach us to eat the lunch brought from home.* (Aldi's father)

Correspondingly, Vian also specified:

*[Q: Whom do you want to go to park with?] With cousins, with family.*

### 3.4. Theme 4: The Need for a Safe Space

A safe space is an environment that provides not only physical safety but also a sense of freedom from being subjected to discrimination, criticism, harassment, or any other emotional harm. It was noted that the behavior of the people around could make children feel unsafe and incapable. Children could not fully participate and express themselves when they feared that they might be judged or bullied, thus impacting belongingness and safety. For example, prior to the study, Vian, Aldi, and Fafa used to refuse to play outside out of fear of being bullied and having low self-esteem. Vian's mother added that her son was eager to interact with other children in the play spaces, if only he felt welcome.

*… because Vian is a shy boy, he would only play with those other children who welcomed him.* (Vian's mother)

Furthermore, some of the children in this study could not fully use the facilities and several pieces of play equipment provided in the play spaces. The observational data revealed how Abdul was unable to climb up the bridge of the jungle gym while the others could; thus, he was teased by another child. In addition, the uneven ground surface made it difficult for Abdul to walk to access different parts of the park, and the other children teased him for moving too slowly. This instance exemplified how a negative social attitude could stem from inadequate physical features.

Moreover, most parents in this study expressed their concerns for the safety of the play equipment and other park/playground facilities for their children, such as the absence of a fence around the fishpond, hard surfaces, and rusty play equipment that posed a risk of infection. Inadequate safety would force the caregivers to be on high alert and made them reluctant to take their children to the play space.

*In terms of safety, a park that has a pond should have a fence to prevent the child from falling into the pond. For example, in Mas Kemambang park, the pond does not have a fence; further, we do not know how deep the pond is. Personally, as much as possible, there needs to be a protecting/safety fence to prevent the child from falling and parents do not have to constantly be cautious.* (Zaki's mother)

The presence of an adult/caregiver served an important role in providing support and security for the children during outdoor play, including giving verbal encouragement, practical physical assistance, and protecting the children from danger. Encouragement and support could substantially impact a child's confidence, thereby being a great facilitator during play in the public play spaces.

*Fafa needs to be motivated to attempt something that is scary for him, only then he would be brave enough to do so. Initially when he played on the playground, he was afraid to do so; but after being motivated, he became sufficiently brave to try it, and finally, he could enjoy playing on that.* (Fafa's mother)

It was also seen in the observational video where Fafa managed to climb the semicircular bridge of the jungle gym for which he initially displayed fear, with physical assistance and words of encouragement from the teachers.

### 3.5. Theme 5: How a Play Space Should Be

According to the interviews, most participants expressed a need for play space improvements. As implied by Ahmad's statement below, the current play spaces were not aligned with preferences:

*(Interviewer: What kind of a park does Ahmad want to visit?) … that only exists in my imaginary world.* (Ahmad)

Most children and some parents stated that they preferred more variations and challenges in the play equipment.

*A park that has various types of play equipment will be better because Abdul gets bored quickly. Like when he would play the swing, he would get bored eventually; thus, we need to divert his attention by taking him to eat or do other things.* (Abdul's mother)

*Make a (park that has) pool, a playground, and a ball bath, and toy houses, toy motorbike, and many more so that more people will come, and it is not boring. Slides, extreme attractions. The ones for adults, for 11 years and above, are tall ones (play equipment). And something for the little children to enjoy.* (Aldi)

In addition, the children wished for the presence of animals and trees/nature.

*(A good park) has many play equipment… flowers… swings. There are trees and cool (air).* (Alia)

Moreover, the parents primarily brought up issues relating to safety, moral/religious values, and educational components of the play spaces. In terms of safety, they suggested softer surfacing, fences, rust-proof play equipment material, and measures to prevent a child from going missing.

*Regarding safety, the play equipment should be provided that is truly safe for the children. Occasionally, I see that the sides or the bottom of the playground are made of concrete. Personally, the soil is better, because it is softer than the bricks that are hard and dangerous if the child falls.* (Vian's mother)

Some of the parents in this study also emphasized the importance of considering moral and religious values when designing and building public play spaces. For instance, a park should not combine play areas with those for teenagers or adults, as elaborated by Vian's mother below:

*What I do not like is that currently, the parks are more teenager-centered; thus, I have reduced the frequency of taking Vian to play in the park. Because personally, there are many things in the park that contradict with what has been taught, and the children easily absorb what they see. … In the park, there should at least be regulations prohibiting dating…. Nowadays, it is especially difficult to find a designated children's play area; hence, I really limit taking my children outside to play.* (Vian's mother)

In addition, some parents preferred that the public play spaces also provide educational play equipment or facilities, so that their children can learn while playing and having fun. Learning about traffic and Indonesian culture and traditions was among the examples given by the parents regarding incorporating educational play components.

## 4. Discussion

Utilizing hermeneutic phenomenological inquiry, this study is aimed at exploring the experiences of children with disabilities in accessing and using the public play spaces. The results revealed that these children visited the public play spaces in search of fun in much the same way as other children as highlighted by a previous research [6, 38, 39]. In the quest for fun, they explored different play equipment, played games with their friends or siblings, or simply enjoyed nature, while experiencing the feeling that time was slipping away. Nonetheless, providing a safe space is important so that the children can fully participate with confidence and a sense of autonomy [40]. The feeling of safety and self-efficacy can be experienced when children feel belong and secure in a group or a space [18]. However, the physical and social environments often posed barriers to feeling safe in children with disabilities and their caregivers. The threat of physical and emotional harm, such as unsafe or inadequately designed play equipment, along with negative attitudes of other users, could hinder participation [6, 20, 41, 42]. Negative social attitudes can also be barriers due to the stigma of disabilities [43] or as a result of physical barriers in the design elements that prevent children with disabilities from using the play equipment [16]. Due to physical barriers, disabled children often relied on adult assistance. Therefore, social participation and their sense of autonomy would be limited, as the fear of limited skill sets and subsequent teasing could result in avoidance of play spaces [44]. In this study, Vian's, Aldi's, and Fafa's parents stated that their children used to refuse to play outside, which was caused by having low self-esteem and the trauma of being bullied.

It has long been established in the United Nations Convention on the Rights of the Child (UNCRC) that to feel safe is a fundamental right of all children, along with the right to fully participate and be included in the community [1]. Yet over three decades since the United Nations Convention on the Rights of the Child, disabled children still face obstacles in their pursuit of this right to safety and freedom from adverse social attitudes [45]. Consequently, these children are unable to enjoy their rights to play equitably, as the fulfillment of these rights relies on feeling safe in such environments [46]. Children feel included when being invited to play by their peers and when receiving equal treatment during play [47]. For this reason, it is vital to remove physical accessibility barriers and hazards, while facilitating equal play opportunities, social interaction, and inclusion. This could be achieved through involving or consulting children with disabilities and their caregivers, or individuals who have close contact with disabled children (e.g., disability organizations/advocates and occupational therapists), regarding the design and planning of play spaces [17, 48]. Participatory or codesign approach is potentially useful to facilitate this process [49].

Apart from the physical design aspect, it is important to promote a welcoming atmosphere through reducing negative social attitude toward children with disabilities. Increasing awareness of disability and social inclusion among children and youth is one way to create a safe play space environment, as the level of knowledge of and exposure to disability often significantly impacted how children viewed and treated their peers with disabilities [50, 51]. Intervention programs to tackle these issues are necessary since children's adverse attitude and low acceptance usually persist without such supportive programs [52]. Moreover, it was demonstrated in a recent study that segregation and exclusion still occur in inclusive playgrounds, indicating that the scope of the issue transcends the physical aspect of the environment [18]. Furthermore, many authors argued that although inclusive policy implementation is important, that alone is not sufficient to achieve social inclusion of children with disabilities [52]. The potentially effective intervention approaches proposed by the existing literature include dismantling stereotypes and creating awareness of the barriers encountered by those living with disability. Such approaches are delivered through the combination of different formats such as simulations, multimedia, and curriculum-based interventions, all of which involve contact with a person who has disability [53]. Occupational therapists or other rehabilitation professionals, educators, and policy makers are in optimal positions to collaborate on developing successful intervention programs.

This study is further aimed at discussing future playground recommendations from the perspective of children with disabilities and their parents. According to the findings, some qualities that the participants sought are related to variations and moderated risks/challenges in the play equipment, the presence of animals, trees/natural surroundings, safety measures, consideration of moral/religious values, and the incorporation of educational components. Despite the importance of safety, risks and challenges are desirable and important elements in play for children [6, 17, 38, 39]. Mainly focusing on the safety standards led to a low play value and an unappealing play environment [6, 43]. The lack of risky play opportunities made play spaces less attractive, thus potentially making them unusable [6]. Other researches highlight the need to include play opportunities that provide appropriate levels of risks and challenges that cater for all ability ranges and developmental levels [43, 54]. Nonetheless, the parents in this study attributed great importance to the safety measures (e.g., softer surfacing, fences, and rust-proof play equipment material) of the play spaces. The presence of safety features influenced the parents' willingness to visit and stay in play spaces [16]. Previous studies suggested that this applies to other parents or families regardless of the child's abilities [55]. Therefore, it is important to consider and balance the provision of risky play opportunities and elements of safety. It might be necessary to provide informational signage containing guidance on how to use the equipment safely, how parents can support their children, and potentially dangerous areas to avoid [17]. The signage should include multiple formats, such as photos/diagrams, braille, and audible descriptions. Additionally, a trained staff or volunteer could be appointed to provide supervision while promoting the independence of children with disabilities [17, 18].

Furthermore, the presence of animals and moral/religious values were themes that have scarcely been discussed in the existing literature relating to the provision of usable public play spaces. However, the studies on children-animal interaction and animal-assisted therapy for children with neurodevelopmental disorders found that the presence of animals had positive benefits on children's social–emotional and cognitive development, as well as their motivation and engagement [56, 57]. Thus, it is surmised that including this component when building a public play space could potentially contribute to positive results in children's participation. Regarding the moral/religious values, it is believed that occupations and moral values are fundamentally interrelated, as occupations have aesthetic and moral dimensions, and they help to develop meanings (intellectual, aesthetic, and moral) [58]. For this reason, it is important to build public play spaces that fit the context or values of the overall community of origin.

## 5. Recommendations for Occupational Therapists Who Are Involved with Playground Design

This study provided insight regarding personal and environmental factors that affect the performance and engagement of children with disabilities in public play spaces, which can aid occupational therapy practitioners in clinical decision-making to promote participation and occupational performance. As occupational therapists, one way to foster participation and inclusion of children with disabilities is by cooperating with local authorities, disability organizations, or educators in initiating disability awareness intervention programs for children and youth. Furthermore, the findings suggest important points of consideration when planning and designing usable, inclusive play spaces, in which occupational therapists could play an active role.

In relation to the rights of individuals with disabilities, despite the existence of international policies that uphold these rights, legislation at the national and local levels is essential in facilitating social change for the provision of usable and inclusive play spaces. Therefore, to facilitate change in the national or local policies, occupational therapists are encouraged to act as a “voice” in public discourse. We can speak up jointly in the media or public arenas about the significance of outdoor play in children's lives and societal conditions that result in the constraints and exclusion experienced by children with disabilities in the public play spaces.

## 6. Methodological Considerations and Future Research

The limitations of this study included limited access to direct interaction with the participants that might have restricted the ability to build rapport and capture subtle facial expressions or body language. Transferability is also limited to the characteristics of the participants in this study that excluded the children with severe physical and hearing impairments. Thus, more heterogeneity in the study participants is recommended for future investigation. Additionally, investigating play spaces in different countries and regions to understand the variability in accessibility and usability for children could generate valuable insights. Furthermore, as a recommendation, participatory action research involving children with different abilities and the professionals across various disciplines in building and designing inclusive play spaces could have a strong impact on the desired change. There is also a need to understand the perspectives of the children who do not access or use public play spaces to determine the causes of their nonuse.

## 7. Conclusions

The public play spaces have meaningful values for children with disabilities and their families, as they offer a multitude of opportunities, including play, fun, exploration, social participation, interaction with animals, access to nature, and learning. However, for children to fully participate with confidence and a sense of autonomy, they need to feel safe, to be free from physical and emotional harm. Nonetheless, regardless of their abilities, children generally craved risky play opportunities, namely, the challenge, complexity, height, or speed elements. Therefore, it is vital to remove physical accessibility barriers and hazards, while promoting equal play opportunities by providing appropriate levels of risk and challenge that cater to children with different abilities and developmental levels. This study further proposed the utilization of participatory or codesign approach involving children with disabilities and their caregivers, rehabilitation professionals, or other individuals who work with disabled children (e.g., disability organizations/advocates), in designing and building future play spaces. Lastly, we highlighted the necessity of awareness-raising intervention programs to bolster the inclusion of children with disabilities in public play space settings.

## Figures and Tables

**Table 1:** Demographic information of the child and adult participants.

Child's pseudonym	Sex and age of the child	Diagnosis	The adult's relationship with the child and age	The adult's occupation
Fafa	Male, 10 years	LD^∗^	Mother, 38 years	Entrepreneur
Alia	Female, 10 years	LD	Mother, 41 years	Entrepreneur
Abdul	Male, 7 years	ADHD^∗∗^	Mother, 40 years	Civil servant
Zaki	Male, 12 years	ASD^∗∗∗^	Mother, 37 years	Entrepreneur
Aldi	Male, 11 years	Low vision	Father, 43 years	Teacher
Vian	Male, 12 years	Epilepsy	Mother, 31 years	Housewife
Ahmad	Male, 9 years	ADHD and epilepsy	Mother, 37 years	Entrepreneur

^∗^Learning disabilities. ^∗∗^Attention-deficit/hyperactivity disorder. ^∗∗∗^Autism spectrum disorder.

## Data Availability

Access to raw data is restricted by virtue of legal and ethical concerns, notably concerning third-party rights and privacy. All other information data is embedded within the manuscript.

## Appendix

### A. Guide to Interviews with Children with Disabilities

(1) Play experiences
  - Tell me about your favorite play activities
  - Tell me about what it is like when you play outside
(2) Park/playground experiences

- Tell me what it is like when you play on the parks/playgrounds (how did you feel?)
- Which parts of the park/playground did you go to, which equipment did you use
- With whom and how often you visit the parks/playgrounds
- With whom do you usually play with on the parks/playgrounds
- What are your favorite and least favorite activities and pieces of equipment on the parks/playgrounds

(3) Tell me about your dream parks/playgrounds

### B. Observation Record

Participant:

Date:

(A) Descriptive notes
  1. Occupations/situations/activities
  2. Who are involved?
  3. Interaction between people and influence between each other and the ongoing activity
  4. What artifacts are used (and not used)?
  5. Interactions that occurred in relation to environmental contexts (does the environment change/restrict/facilitate?)
  6. What does not happen? (notable nonoccurrences)
(B) Reflective notes
